# Role of CXCL10 in Spinal Cord Injury

**DOI:** 10.7150/ijms.76694

**Published:** 2022-11-14

**Authors:** Xinyu Qiao, Wei Zhang, Weijiang Zhao

**Affiliations:** 1 Wuxi School of Medicine, Jiangnan University, Wuxi 214122, Jiangsu, China; 2 Department of Pathogen Biology, Guizhou Nursing Vocational College, Guiyang, China; 3 Cell Biology Department, Wuxi School of Medicine, Jiangnan University, Wuxi 214122, Jiangsu, China

**Keywords:** CXCL10, Spinal cord injury (SCI), CXCR3, Inflammatory, Secondary damage, Complications

## Abstract

Spinal cord injury (SCI) results in acute inflammatory responses and secondary damages, including neuronal and glial cell death, axonal damage and demyelination, and blood-brain barrier (BBB) damage, eventually leading to neuronal dysfunction and other complications. C-X-C motif Chemokine Ligand 10 (CXCL10) is expressed after the injury, playing multiple roles in the development and progression of SCI. Moreover, the CXCL10 antagonist can restrict inflammatory immune responses and promote neuronal regeneration and functional recovery. In this review, we summarize the structure and biological functions of CXCL10, and the roles of the CXCL10 / CXCR3 axis in acute inflammatory responses, secondary damages, and complications during SCI, thus providing a potential theoretical basis by highlighting CXCL10 as a new potential drug target for the treatment of SCI.

## Introduction

Spinal cord injury (SCI) refers to an injury in the spinal cord, usually caused by external physical impact 1. Traffic accidents and falls constitute the major causes of SCI 2, 3. SCI causes a series of complex pathological changes. It directly causes neuronal damage, cell death, ischemic injury, and inflammation. Also, it leads to complex secondary damages, such as the formation of glial scar composed of reactive astrocytes at locations both proximal and distal to the injury site, and degenerative lesions in the tissue and structure of the spinal cord namely the formation of the cystic cavity 4-9. Glial scars and cystic cavities cause poor regeneration of myelin and axons, finally leading to permanent neurological deficits characterized by long-term sequelae and complications, including loss of motor functions, vegetative nervous system dysfunctions, neuropathic pain syndromes, and even cognitive impairment 4, 10-12.

The effects of SCI on patients result in a loss of self-care and an increased risk of death 13. The acute mortality rate during hospitalizations ranges from 4% to 17%. After leaving the hospital, the annual mortality rate remains high: 3.8% in the first year and 1.6% in the second year after the injury, and it remains at 1.2% thereafter 14. Given the serious long-term effects of SCI, it is critical to develop effective treatments for those cases that cannot be prevented.

The secondary damage is defined as the pathological cascade caused by the primary injury, which has been the focus of research in the past several decades. Post-traumatic inflammation is considered to be an important influencing factor of secondary damage. Inflammatory factors and inflammatory factors-mediated inflammatory responses can significantly promote or inhibit the repair process of SCI during acute, subacute, and chronic phases 15-18. The binding of chemokines to their receptors is an essential component of many secondary inflammatory mechanisms during these phases 19. In particular, CXCL10, known as IP-10 (Interferon-gamma inducible Protein 10 kDa), belongs to the ELR^-^ CXC subfamily of chemokines. CXCL10 functions by binding to C-X-C chemokine receptor type 3 (CXCR3), and CXCL10 / CXCR3 axis plays a pro-inflammatory role in immunoreaction 20, 21.

CXCL10 exerts a wide range of functions in several main pathophysiological changes in SCI, such as inflammatory cell recruitment, angiogenesis inhibition, apoptosis promotion, neuronal loss, axonal injury, neuropathic pain, and impaired motor function recovery 22-25. We therefore speculate that targeting CXCL10 and its receptor is of great potential therapeutic value in the management of SCI.

We here briefly review the structure and biological functions of CXCL10 and elucidate the critical role of CXCL10 in the pathogenesis of inflammatory responses and secondary damages to SCI, which may provide a theoretical basis for CXCL10 as a potential biomarker and therapeutic target of the central nervous system (CNS) inflammatory responses.

## CXCL10 and its Receptor CXCR3

### CXCL10

Chemokines, with a molecular weight ranging from 8 kDa to 12 kDa, are involved in a wide range of physiological and pathological processes in the CNS. Based on the presence of cysteine moieties at the N-terminal sequences of a protein, chemokines can be divided into four subgroups (C-, CC-, CXC-, and CX3C-, defined based on four conserved cysteine residues that form disulfide bonds), which act on G protein-coupled chemokine receptors 26, 27. In the adult CNS, chemokines and their receptors are involved in developmental, physiological, and pathological processes, such as inducing cell migrations, promoting cellular interactions, activating intracellular signaling pathways, and maintaining CNS homeostasis 28.

CXCL10 was initially identified as a chemokine induced by IFN-γ 29. It belongs to the CXC subfamily of chemokines, which contains four conserved cysteine residue motifs linked by intervening amino acids between the first two conserved cysteines 27. According to the presence or absence of the acid-leucine-arginine (Glu-Leu-Arg, ELR) motif in the N-terminal region, CXC chemokines can be divided into two subgroups: ELR^+^ CXC chemokines can promote angiogenesis, whereas ELR^-^ chemokines, such as CXCL10, can inhibit angiogenesis 20, 30.

CXCL10 can be secreted by various types of cells, including immune cells such as T lymphocytes, neutrophils, eosinophils and monocytes. It can also be secreted by stromal cells, including thyroid cells, splenocytes, endothelial cells, fibroblasts, and keratinocytes 31-34. TNFα, IFNα, β and γ are all inducers of CXCL10 35-37.

### CXCR3

**Figure 1:**
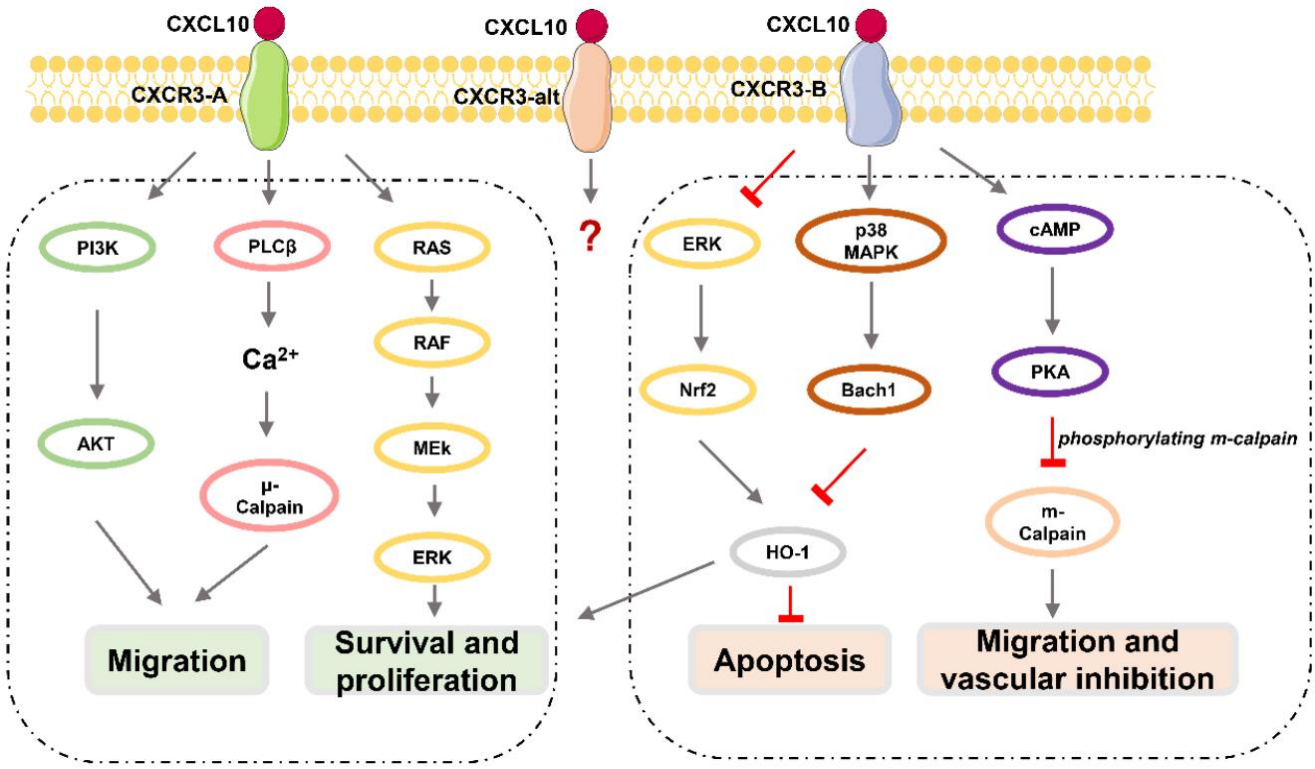
** Downstream effects of CXCL10 / CXCR3 signaling pathway.** CXCR3-A promotes proliferation, cell survival, chemotaxis, and invasion, while CXCR3-B mediates growth inhibition, apoptosis, and vascular inhibition.

CXCR3, also known as GPR9 or CD183, is a G protein-coupled seven-transmembrane receptor that can also be expressed in immune cells such as CD4^+^ T lymphocytes, CD8^+^ T lymphocytes, and NK cells, and in stromal cells such as endothelial cells, glomerular mesangial cells, trophoblasts, and keratinocytes 38, 39. CXCR3 can be divided into three subtypes (Figure 1): CXCR3-A, CXCR3-B, and CXCR3-alt 40-43, among which CXCR3-A and CXCR3-B are expressed in neurons 41, 44. CXCR3-A signaling promotes cell migration and invasion via the PLCβ3 / μ-calpain signaling pathway in prostate cancer 45. CXCL10 also binds to the CXCR3-A subtype, fully induces Gαi activation and extracellular signal-regulated kinase (ERK) 1 / 2 phosphorylation, and partially induces the recruitment of β-arrestin protein 40. CXCR3-A mediates chemotactic responses via MAPK and PI3K / AKT signaling pathways in human airway epithelial cells 46. Activation of CXCR3-B can inhibit angiogenesis or proliferation and promote apoptosis 47. CXCL10 acts as an angiostatic agent, via CXCR3-B signal-mediated PKA phosphorylation of m-calpain to prevent endothelial cell motility 48-51 or via activation of p38/MAPK activation in human microvascular endothelial cells 52. CXCR3-B mediates growth inhibition or apoptosis via p38/MAPK activation following the downregulation of heme oxygenase 1 (HO1) and the translocation of Bach1 and Nrf2 in human renal cancer cells and breast cancer cells 53. Although CXCL10 can somewhat induce the activation of CXCR3-alt, its role in this process is unclear 54.

### The Basic Functions of CXCL10 / CXCR3 axis

CXCL10 has a variety of biological functions such as chemotaxis, differentiation, activation of immune cells, regulation of apoptosis, and neovascularization inhibition through the CXCL10 / CXCR3 axis.

One of the basic functions of CXCL10 / CXCR3 axis is that activation of the CXCL10 / CXCR3 axis activates the inflammatory cells and orchestrates inflammatory cell migration. For example, it activates microglia and directs them to the site of injury 42, 55, 56. And it forms an amplified feedback loop in activating the inflammatory cells. In CD4^+^ T lymphocytes, CXCR3 is induced by T cell receptor (TCR), enhancing the production of IFNγ and TNFα, which stimulates various cells to secrete CXCL10, thus forming an amplified feedback loop and perpetuating the immune cascade 57, 58. Besides CD4^+^ T lymphocytes, CXCL10 also stimulates the directional migration of CD29^+^ T lymphocytes and monocytes, as well as potentiates T lymphocyte adhesion to endothelium 59. In addition, CXCL10 directs CD8^+^ T lymphocytes and NK cells recruiting in CNS immune response 60-63. Besides T lymphocyte recruitment, CXCL10 induces eosinophil chemotaxis, which can be blocked by the anti-CXCR3 monoclonal antibody, via CXCR3 inactivation on eosinophils 64. Therefore, CXCL10 plays an immunoregulatory role in innate and adaptive immunity.

In addition, CXCL10 functions as a suppressor of neovascularization by inhibiting the expression of various angiogenic factors in vivo, including VEGF a and c, and MMP13, and directly inducing endothelial cell apoptosis. In vitro, CXCL10 inhibits endothelial differentiation into tubular capillary structures in a dose-dependent manner, whereas it does not affect endothelial cell growth and migration 65, 66.

## Expressions and Roles of CXCL10 in the CNS

The CNS consists mainly of neurons and glial cells, and a small amount of vascular and connective tissues. Neurons undertake the main functions of the nervous system, and the glial cells (including astrocytes, oligodendrocytes, and microglia) participate in the formation of the blood-brain barrier (BBB), controlling immune responses in a dominant manner, and participating in nerve repair and regeneration.

Under physiological conditions, CXCL10 expression in the CNS is not detectable. However, the CNS cells can synthesize chemokines and bind to them via receptors on their surface, thus producing chemotaxis and other functions 67, 68.

### Expressions and Roles of CXCL10 in Neurons

The immune response of the nervous system is predominantly undertaken by glial cells. However, in some cases, like viral infection, neurons expressing CXCL10 trigger inflammatory responses in the absence of glial cells. Viral dsRNA binds to TLR receptors on nerve cells, leading to a virus-mediated innate immune response that results in the expression of the inflammatory cytokines IL6 and TNFα, chemokines CCL5 and CXCL10, and the antiviral molecule IFNβ 69. CXCL10 can be toxic to cells in the CNS. For example, CXCL10 can directly cause astrocyte death along with indirect neuronal death in HIV1 Nef protein-mediated neuronal cell death 70.

In human fetal neuron/astrocyte co-culture and human NT-2 neurons, CXCL10 was sufficient to induce apoptosis. In addition, a surge in CXCL10 expression under a variety of pathological conditions can lead to neuronal death and loss. CXCL10 can serve as a functional biomarker of human cerebral malaria (HCM) mortality and promote apoptosis of brain neurons and glial cells 71, 72. In HIV1-infected patients, CXCL10, in combination with HIV1, can synergistically enhance neuronal toxicity, which can be inhibited by blocking CXCR3 and its downstream MAPK signaling pathway 73.

The neurotoxic mechanisms involving CXCL10 are as follows: CXCL10 induces apoptosis through the mitochondria-dependent pathway by increasing the intracellular load of calcium released from the endoplasmic reticulum, leading to the mitochondrial release of cytochrome C (Cyt C) and cleavage-activation of caspase3 74-76.

### The Expression and Roles of CXCL10 in Glial Cells

In the CNS, chemokines, expressed primarily by glial cells, recruit lymphocytes and phagocytes to inflammatory and infected areas and promote the immune response. Astrocytes and microglia exert a definite role in CXCL10 secretion. Microglia are resident macrophages in the CNS 77. They provide immune defense and contribute to multiple functions during CNS development and maturation. Microglia secrete CXCL10 under a variety of conditions. For example, Borrelia burgdorferi, the pathogen of neuroborreliosis, can induce increased expression and secretion of CXCL10, which may be involved in a stronger activation effect on neuroinflammation induced by dead Borrelia burgdorferi 78.

Stimulation of human microglia by LPS or IFNβ produced during spirochete infection results in enhanced transcription of CXCL10 and microglia-derived CXCL10 secretion 79-82. IFNβ promotes CXCL10 expression in microglia through the JAK-STAT signaling pathway and LPS can bind to TLR4 receptor and induce nuclear translocations of IFN Response factor 3 (IRF 3) or NFκB to promote CXCL10 expression in microglia 83-86.

Astrocytes also play a role in secreting CXCL10 in a variety of diseases. CXCL10 attracts phagocytes and promotes inflammatory responses. And human astrocytes and microvascular endothelial cells produce specific chemokines including CXCL10 to attract phagocytic cells and promote an inflammatory response in Borrelia burgdorferi infection 87. Astrocytes expressing CXCL10 enhance viral infection and neuronal damage through the binding of TLR3 receptors to viral dsRNA 88. Astrocytes are the main sources of CXCL10 in Zika virus infection, Japanese encephalitis virus infection, and HIV-associated encephalitis 67, 73, 89, 90. Astrocytes are also a major source of CXCL10 in neurodegenerative diseases. During the development of AD, the expression of CXCL10 is significantly increased in astrocytes, where it activates the ERK1 / 2 signaling pathway in mouse cortical neurons and forms a neuron-glial interaction 43, 76, 91. Astrocytes are also the primary source of CXCL10 in the pathogenesis of CNS immune diseases, such as multiple sclerosis (MS) 82, 92-96.

CXCL10 binds to CXCR3 expressed by microglia and astrocytes, and the effect of CXCL10 on microglia and astrocytes is mainly focused on their migration promotion effects 97, 98. CXCL10 binds to CXCR3 to induce calcium influx and electrophysiological responses, resulting in the recruitment of distal microglia via chemotaxis 97, 99, 100. However, the proliferation of microglia is not affected 42. In addition, CXCL10 / CXCR3 axis is closely related to the activation of glial cells, and CXCR3 deficiency significantly weakens the activation of microglia and astrocytes 101. Moreover, CXCL10 also induces apoptosis in a dose-dependent manner in glial cells, in which it induces the expression and activation of caspase 3 and 7, thus increasing the percentage of apoptotic human neuroglia cells from 12% to 40.6% 102.

Oligodendrocytes function as myelin-forming cells in the CNS, wrapping around multiple nerve fibers to form the myelin sheath. However, there are few studies on CXCL10 secreted by oligodendrocytes. It has been reported that oligodendrocytes can express a variety of chemokines including CXCL10 in the presence of IFNγ 103. Therefore, most studies focus on the role of CXCL10 on oligodendrocytes in myelination. Some studies demonstrated that CXCL10 expression is highly upregulated by IL1β and IFNγ, in hypertrophic astrocytes surrounding active MS lesions, and CXCL10 finally interferes with myelin formation by acting on CXCR3 receptors on oligodendrocytes 92, 104-106.

## The Role of CXCL10 in SCI

### Basic Pathological Manifestations of SCI

**Figure 2:**
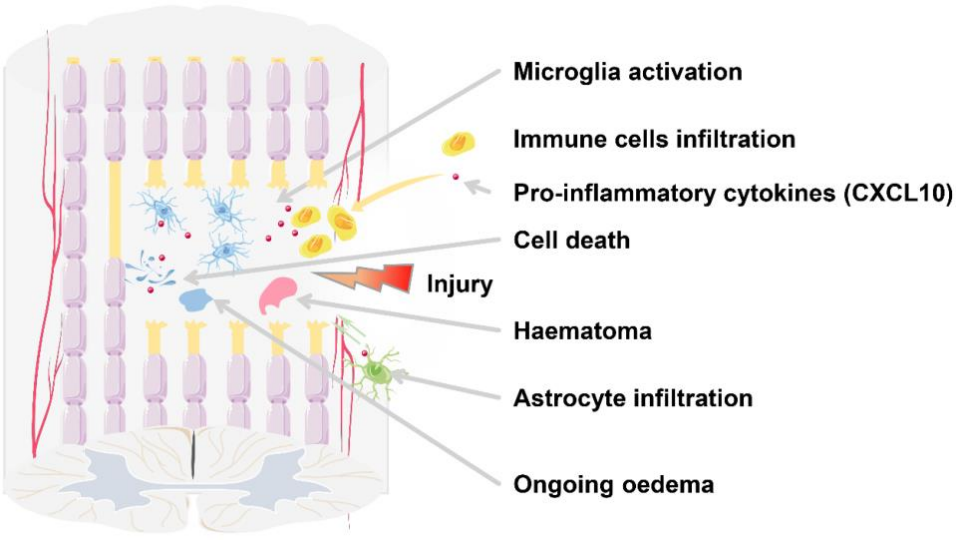
** SCI results in secondary injuries, as well as local and systemic complications.** In the lesion core, the injury leads to neuronal death, axonal damage and demyelination, and other complications.

According to the time course, SCI can be divided into acute (0-48 hours), subacute (48 hours-14 days), intermediate (14 days-6 months), and chronic (>6 months) phases 107. The acute phase is characterized by bleeding, edema, inflammatory cell infiltration, the release of cytotoxic products, and cell death (Figure 2). After SCI, the reduced sympathetic tone and autonomous blood flow modulation mechanism cause a significant decrease in the spinal cord blood flow (SCBF) and the mean systemic arterial blood pressure (ABP), leading to ischemia at the injury site, which can last for days or even weeks 108, 109. Moreover, the trauma directly leads to the displacement or rupture of the spine, resulting in compression or damage to the spinal cord and the destruction of the blood-spinal cord barrier (BSCB) environment, exposing the damaged spinal cord tissue to peripheral cytokines and blood cells 110. As a result, local cells die due to changes in cell permeability, initiation of pre-apoptotic signaling, and ischemia caused by vascular damage 111, 112. Dead cells release ATP, DNA, and K^+^, which activate the microglia to secrete pro-inflammatory cytokines including CXCL10, causing local inflammatory cell infiltration.

Microglia and endothelial cells play a role as antigen-presenting cells (APC) during the initial stage of injury. T lymphocyte infiltration preceding the bulk of monocyte influx and macrophage activation continues throughout the acute to subacute phase 113. Inflammatory cells accumulate at the injury site, releasing cytokines such as TNF, IL1, IL18, CCL2, and MMP9, and cytotoxic molecules like glutamate, which have neuroexcitatory toxicity and can cause spinal cord white matter hypoxia and traumatic injury 114-119. In particular, it is observed that the infiltrating macrophages develop into M1 macrophage subsets and express pro-inflammatory cytokines such as CXCL10 and IL12p70 120.

In the later acute phase to subacute phase, progressive edema exacerbates the post-injury microenvironment. In this case, the inflammatory cells stay in the spinal cord and continuously intensify the inflammatory response from the acute to subacute phase, resulting in further edema of multiple adjacent spinal segments. In the subacute phase, edema, combined with vascular thrombosis and vasospasm, causes ischemia and exacerbates infiltration of inflammatory cells, further leading to cell death. Cystic cavities containing extracellular fluids, small bands of connective tissue, and macrophages start to form as a consequence 121, 122. Astrocytes proliferate and deposit into the surrounding area of the lesion core. During the intermediate phase to the chronic phase, axons continue to degenerate. And mature glial scars and cystic cavities further inhibit axonal regeneration and cell migration 123.

### SCI-induced CXCL10 Changes in the Spinal Cord, the Cerebrospinal Fluid and the Blood Serum

The changes in the CXCL10 expression level show a certain correlation with the process of secondary damages. In humans or a variety of animal models like those in rats or mice, increased CXCL10 expression levels can be detected in the core area of the spinal cord, serum, and cerebrospinal fluid (CSF).

As reported by McTigue et al, the mRNA expression of CXCL10 can be detected from 6 hours to 28 days in the lesion core of rats after SCI, with the peak identified at 6 hours post-injury 124. Although the expression level of CXCL10 decreases after 6 hours post-injury, it is still significantly high at 12 hours post-injury and is attenuated to the control level subsequently 124. In particular, the mRNA expression of CXCL10 is visible in the mouse spinal cord 30 minutes post-injury 125. All these reports indicate that CXCL10 is mainly expressed by cells in the lesion core during the acute phase of SCI including microglia, astrocytes, and other inflammatory cells such as NK cells, CD8^+^ T lymphocytes, and macrophages 22, 123, 126, 127. Furthermore, inflammatory cells like microglia in the spinal cord are sufficient to trigger an inflammatory response without the presence of peripheral blood-derived cells in the mouse SCI model 125. Microglia are the main source of CXCL10 in early acute injury, and CXCL10 is co-expressed in microglia and other inflammatory cells, including astrocytes and macrophages 56, 120, 128.

There are few studies about the expression of CXCL10 in CSF. Casha et al reported that CXCL10 significantly increases at 6 h post-injury, and then reaches the peak within 2 days in the acute phase, followed by a slight decrease at day 3, and a more sustained increase between days 4 and 7, exhibiting a bimodal release mode 118.

The trend of CXCL10 concentration changes in the peripheral blood is relatively consistent with that in the CSF. Hassanshahi et al reported that the concentration of CXCL10 increases significantly in the acute SCI phase (3-6 hours post-injury), reaches the maximum value after the subacute phase (7 days), and then decreases. Notably, significantly increased CXCL10 concentration can still be detected after the intermediate phase (28 d) until 30 days post-injury when the concentration of CXCL10 is equal to that of the control 129, 130.

To summarize, the level of CXCL10 in the CSF, the peripheral blood, and the lesion core is relatively consistent over time. CXCL10 exhibits a high level in the acute phase post-injury, and the level maintains at higher concentrations in the subacute phase, and remains obvious in the intermediate period, suggesting a certain relationship between the changes of CXCL10 and the secondary damage.

### Roles of CXCL10 in SCI-induced Neuroinflammation

In the acute phase of mice injury, Rice et al reported that the activated microglia of the CNS release a large amount of CXCL10 at 3 hours post-injury, playing a key role in inflammatory cell aggregation 125. CNS-derived CXCL10 performs their function of CD4^+^ Th1 cells recruitment by binding to the CXCR3-A receptor, and then CD4^+^ Th1 cells secrete IL2, IFN, TNF, and other cytokines to participate in the regulation of cellular immunity, enhance macrophage toxicity, regulate the differentiation of CD8^+^ T lymphocytes, and trigger the immune response. In the meantime, the immune cells recruited in response to CXCL10 also release CXCL10, resulting in a cascade reaction 24, 120.

There may be two reasons for the destruction of the BBB caused by CXCL10 in the acute phase. On the one hand, the BBB structure maintains the homeostasis of the CNS and restricts the recruitment of peripheral immune cells into the brain parenchyma composed of brain microvascular endothelial cells, pericytes, and astrocyte endfeet. CXCL10 inhibits the migration of endothelial cells through the CXCR3-B receptor, inhibits DNA synthesis, and promotes endothelial cell apoptosis, thus destroying the BBB 47, 102. On the other hand, CXCL10 drives the aggravation of inflammatory responses and indirectly disrupts the BBB by oxidative stress and neurotoxic molecules 131. This oxidative stress is generated by reactive oxygen species and nitric oxides released by inflammatory cells such as microglia and astrocytes, and neurotoxic molecules including prostaglandin, cyclooxygenase 2, MCP1 and MIP1α, as well as pro-inflammatory cytokines IL6, TNFα, and IL1β 131. Therefore, CXCL10 involved in destruction of BBB in the acute phase, which creates a unique condition for the accumulation of peripheral immune cells, expanding the immune response from the local region to the whole CNS.

The early immune response plays a positive role in the recovery of SCI. To illustrate, immune cells can help to remove tissue debris and trigger the release of various neurotrophic factors 16, 132. However, this autoimmune response gets out of control over time, resulting in secondary degenerative pathologies followed by continued axonal degeneration and glial scar maturation, thus causing detrimental consequences to spontaneous recovery from SCI. A high level of CXCL10 in the CSF during the subacute stages of SCI plays a key role in the secondary degenerative pathologies by restricting revascularization and axonal regeneration, resulting in the loss of the spinal cord tissue 22.

### Potential Underlying Mechanisms for the Role of CXCL10 in SCI

**Figure 3:**
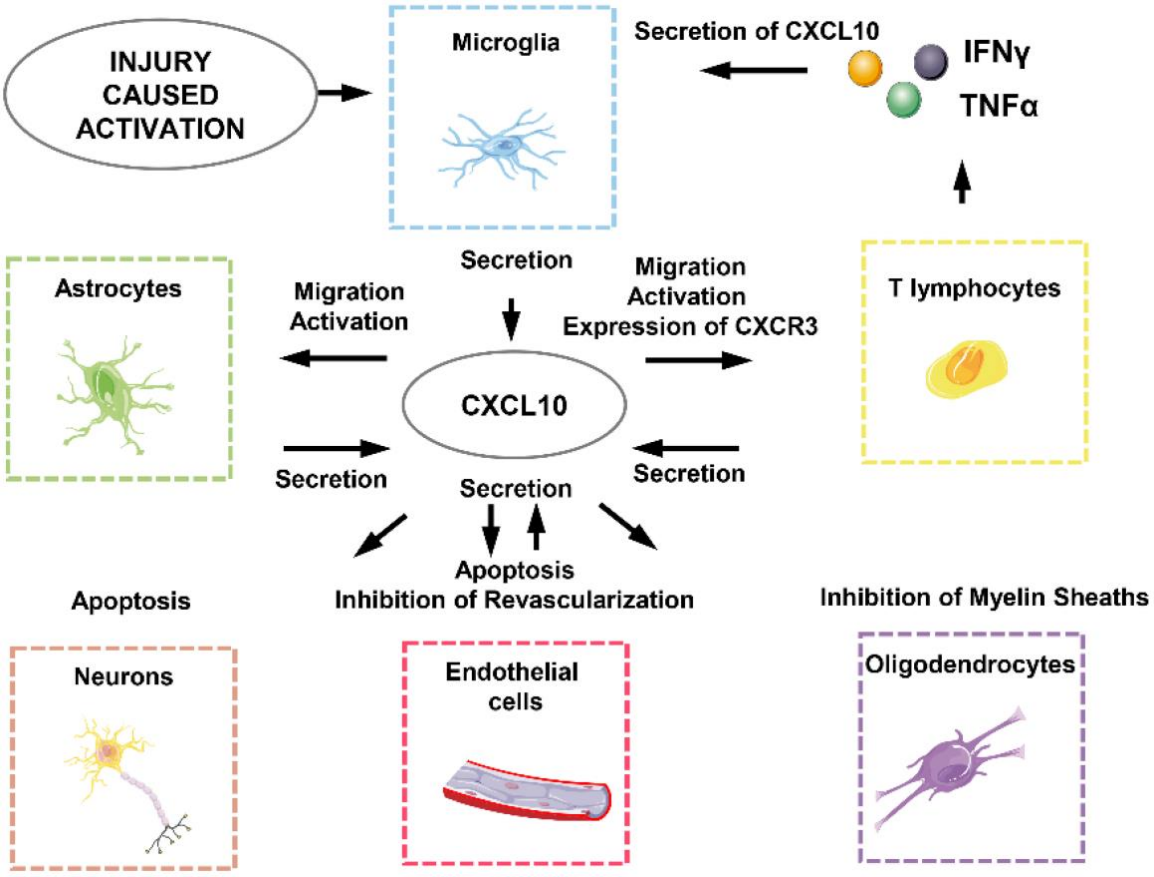
CXCL10-related cellular response in SCI-induced neuroinflammation.

CXCL10 generates biological effects in SCI through interacting directly or indirectly with neurons, astrocytes, microglia, oligodendrocytes, endothelial cells and T lymphocytes (Figure 3) 18. First of all, the effect of local ischemia on necrotic tissue cannot be ignored. The rupture of blood vessels, thromboses and vasospasm, and neurogenic hypotension are all caused by the injury and lead to the loss of blood. At this point, angiogenesis that can relieve the ischemic state is unfortunately directly or indirectly inhibited by CXCL10 24, 133. This means CXCL10 not only directly inhibits differentiation and promotes apoptosis of endothelial cells but also works together with inflammatory cells to involve in subsequent functions 47. CXCL10 can inhibit the expression of various pro-angiogenesis cytokines and receptors such as angiogenesis-associated growth factor VEGF, the receptors Flt1, Flt4, and the thrombin receptor, and the endothelial cell marker CD31 / PECAM1 and the growth factor angiopoietin1. It also causes apoptosis of vascular endothelial cells by oxidative stress as well as a variety of other cytotoxic molecules which plays an indirect role in inhibiting angiogenesis 24, 131.

Secondly, CXCL10 inhibits axonal regeneration. Previous studies have revealed that major phenotypic changes that astrocytes undergo are prerequisites for promoting spinal cord myelination. Astrocytes release CXCL10 to interfere with oligodendrocytes, thereby inhibiting the regeneration of axons of the spinal cord neurons 105. In addition, CXCL10 impairs axonal extension and inhibits axonal regeneration by recruiting inflammatory cells, although the molecular mechanisms are poorly understood 25, 134.

Finally, CXCL10 not only exerts a direct apoptosis-promoting effect on neurons and glial cells but also recruits CD4^+^ T lymphocytes secreting various cytotoxic molecules such as IFNγ and TNFα to induce apoptosis of neurons and glial cells 135-137.

### The Role of CXCL10 in the Complications of SCI

Studies have shown that there is a certain correlation between CXCL10 and neuropathic pain, joint inflammation, dysfunction of the genitourinary system, and pressure sores after SCI.

### Neuropathic Pain

More than 70% of patients suffer from pain after SCI, and about 40% of patients experience chronic nerve pain. The pain can last for years and significantly impact the physical and emotional functions and quality of life 138-140.

Neuropathic pain has not been sufficiently researched to identify the underlying mechanisms. Some studies have shown that nerve root damage may lead to sprouting of spinal cord fibers and activation of primary afferent fibers, causing allodynia and hyperalgesia 141, 142. Several recent clinical studies have found that inflammatory cytokines and chemokines play an important role in the development and persistence of neuropathic pain. Statistical analysis of the relationship between CXCL10 levels and pain in patients with SCI shows that CXCL10 concentrations are positively associated with pain intensity 23. Patients with constant or increased CXCL10 over time have an increased risk of pain at one-year post-injury 23. Therefore, CXCL10 has the potential to serve as a biomarker of pain development in SCI patients. In recent years, many studies have recognized that the interaction between CXCL10 and CXCR3 can participate in neuropathic pain in both the spinal cord and brain 55, 126, 143-148. In rats with SCI, CXCL10 and CXCR3 are significantly expressed in the spinal cord, and CXCL10 / CXCR3 interaction is directly involved in neuropathic pain by regulating the ERK signaling pathway 55.

In addition to its role at the site of injury, CXCL10 / CXCR3 interaction is also involved in the emotional process of pain by aberrant activation of the anterior cingulate cortex (ACC), which is part of the limbic system in the frontal region of the cingulate cortex. The pain involves affective, sensory, and cognitive dimensions 143, and neuronal activity in this structure can lead to an aversive emotion to noxious stimuli 144, 145. One study suggests that CXCR3 can be increased by enhanced interaction between C / EBPα and DNA demethylation post-injury and activated by CXCL10 to maintain neuropathic pain, via the downstream pERK pathway, causing hyperalgesia 146, 147. And the CXCR3 inhibitor AMG487 or (±)-NBI-74330 can significantly improve the pain threshold and can reduce neuropathic pain 55, 148. Another study showed that only CXCL10 increases excitatory synaptic transmission and the risk of pain onset, and it also exacerbates existing pain in mice with SCI. In contrast, CXCL9 and CXCL11, the same subfamily chemokines jointly binding to CXCR3, can enhance both excitatory and inhibitory synaptic transmission without exacerbating pain 126. These combined data suggest an excellent application prospect of anti-CXCL10 means in the treatment of neuropathic pain after SCI.

### Joint Inflammation

Inflammations, heterotopic ossifications, and fibrosis have been observed in the joints following SCI 149. In recent years, most studies on joint complications after SCI focus on heterotopic ossification with an incidence rate of 10-53%. Its clinical presentations include pain, fever, redness, swelling of surrounding tissues, and limited joint movements, followed by the formation of ectopic bones in the periarticular connective tissues 150.

The exact pathophysiology of the joint inflammation remains unclear, while a recent study confirms the role of CXCL10 in this disease 151. CXCL10 increases the migration of F4 / 80^+^ macrophages and CD4^+^ T lymphocytes into the synovium in collagen antibody-induced arthritis (CAIA) mice through ERK activation mediated by CXCR3 151. CXCL10 / CXCR3 axis works synergistically with TLR4 and increases joint inflammations by stimulating the production of osteoclastogenic cytokines in CD4^+^ T lymphocytes 151.

### Dysfunctions of the Genitourinary Systems

The bladder problem (44%) was one of the most common medical problems in patients with SCI 140. SCI blocks the spino-bulbo-spinal reflex pathway, resulting in bladder areflexia with complete urinary retention 152.

CXCL10 is significantly expressed in bladder tissues at 8 weeks and 12 weeks after rat SCI and the poor prognosis with the low recovery of urination function is related to the up-regulated expression of chemokines (represented by CXCL10), other pro-inflammatory factors and anti-inflammatory cytokines 153. CXCL10 can exert an inhibitory effect on the spontaneous recovery of the urination reflex, which might be related to the demyelination of related neuronal fibers.

### Pressure Sores

In clinical practice, pressure sores are one of the common skin diseases which usually occur after SCI. Pressure sores can cause pain and increase the burden of nursing, resulting in a dramatic deterioration of patients' life quality. Pressure sores occur commonly on the buttocks (31%), lateral thighs (26%), sacrum (18%), feet (7%), and ankles (4%) 154. A recent study found that plasma concentrations of CXCL10 are associated with the development of pressure sores before its first occurrence. CXCL10 has the potential to be a biomarker to identify patients at risk for pressure sores progression 155.

## Concluding Remarks

CXCL10 is involved in the process of acute inflammatory responses and secondary damages, and many complications after SCI via CXCR3 receptor 18, 22, 24, 25, 47, 55, 105, 118, 120, 123-127, 129-131, 133-137, 143-148, 150, 151, 153, 155. After SCI, CXCL10 secreted by microglia plays an important role in recruiting and activating inflammatory cells 124, 125. CXCL10 and recruited inflammatory cells work together to aggravate the inflammatory response, inhibit angiogenesis by inhibiting the differentiation of endothelial cells, decrease myelin formation by inhibiting oligodendrocytes, and then promote apoptosis resulting in the progressive loss of tissue at the lesion core 24, 25, 47, 105, 133-137. All these effects lead to the inhibition of spinal cord regeneration. In addition, CXCL10 causes an increased disruption of BBB due to its effects on endothelial cells, resulting in CNS homeostasis disruption, and exacerbating CNS inflammation 47, 102, 131. Furthermore, CXCL10 has proved to be associated with four complications after SCI, including joint inflammation, neuropathic pain, dysfunction of the genitourinary systems, and pressure sores 23, 55, 126, 143-148, 151, 155.

Existing clinical treatments of SCI include a variety of pharmacological and non-pharmacological approaches to alleviate and delay the secondary damage cascade after SCI, including sodium channel-blocking antiseizure drugs Riluzole, the NMDA receptor antagonist magnesium, the antibiotic minocycline, the glycosphingolipid GM1 as a kind of ganglioside with neuroprotective effects, the fibroblast growth factor, the hepatocyte growth factor and G-CSF with anti-inflammatory effects. Although all these drug treatments have achieved certain clinical results, they fail to completely meet the expected therapeutic efficiency for patients 156.

It is undoubtedly a novel means for the treatment of SCI by regulating the immune process via the neutralization of pro-inflammatory factors 157. In both the rat and the mouse model mimicking SCI, CXCL10 antagonist can reduce T lymphocyte infiltration and inhibit neuronal death, thus increasing axonal and blood vessel regeneration and improving functional recovery after SCI 22, 25, 157, 158.

Despite the encouraging results from these aforementioned studies, CXCL10 antagonist alone cannot achieve the desired therapeutic effect. Although CXCL10 can affect regeneration and functional recovery after SCI as a key factor in the microenvironment of the injured spinal cord, several other growth-inhibiting molecules (chemokine and cytokines) also involve in a time-dependent manner 159, 160. For instance, the serum level of TNF-α, MCP-1, IL-1β, IL-2, IL-6, IL-4 and IL-10 is highly time dependent during the progression of SCI and abnormal expression of these cytokines is also related to poor neurological outcomes 161. These findings further emphasize that CXCL10, particularly along with other factors in the microenvironment of the spinal cord, is necessary for SCI deterioration. CXCL10-induced inflammation may also play a neuroprotective role in the CNS. For instance, neurons infected with the neurotropic flavivirus West Nile virus can express CXCL10 to promote adaptive immune responses to clear the virus, casting a new light on the neuroprotective function of CXCL10 60. And other results show that infiltrating blood-derived macrophages displayed an anti-inflammatory beneficial role, and microglia prevented lesion expansion in brain damage and astrocytes to aid CNS axon regeneration in SCI 162-164. It also suggests that inflammation might represent a front-line defense against CNS damage. Furthermore, most results of CXCL10 antagonist currently available have been conducted using animal models with a lack of evidence in human beings, and some specific issues remain to be solved. For instance, the most appropriate therapeutic time window needs further evaluation. Different spinal segments with injury exhibit differential responses among individuals, showing different expression levels of the inflammatory cytokines. Taken together, personalized treatment strategies are still needed to optimize the treatment via targeting CXCL10 signaling pathways and the CXCL10 antagonist treatment should be combinedly utilized with other therapeutic means.

## References

[1] Eckert MJ, Martin MJ (2017). Trauma: Spinal Cord Injury. Surg Clin North Am.

[2] Kumar R, Lim J, Mekary RA, Rattani A, Dewan MC, Sharif SY (2018). Traumatic Spinal Injury: Global Epidemiology and Worldwide Volume. World Neurosurg.

[3] Spinal Cord Injury (SCI) Facts and Figures at a Glance J Spinal Cord Med. 2016; 39: 370-1.

[4] Hagg T, Oudega M (2006). Degenerative and spontaneous regenerative processes after spinal cord injury. J Neurotrauma.

[5] Shi R, Pryor JD (2002). Pathological changes of isolated spinal cord axons in response to mechanical stretch. Neuroscience.

[6] Schnell L, Fearn S, Klassen H, Schwab ME, Perry VH (1999). Acute inflammatory responses to mechanical lesions in the CNS: differences between brain and spinal cord. Eur J Neurosci.

[7] Saville LR, Pospisil CH, Mawhinney LA, Bao F, Simedrea FC, Peters AA (2004). A monoclonal antibody to CD11d reduces the inflammatory infiltrate into the injured spinal cord: a potential neuroprotective treatment. J Neuroimmunol.

[8] Demjen D, Klussmann S, Kleber S, Zuliani C, Stieltjes B, Metzger C (2004). Neutralization of CD95 ligand promotes regeneration and functional recovery after spinal cord injury. Nat Med.

[9] Emery E, Aldana P, Bunge MB, Puckett W, Srinivasan A, Keane RW (1998). Apoptosis after traumatic human spinal cord injury. J Neurosurg.

[10] Rowland JW, Hawryluk GW, Kwon B, Fehlings MG (2008). Current status of acute spinal cord injury pathophysiology and emerging therapies: promise on the horizon. Neurosurg Focus.

[11] Fawcett JW, Asher RA (1999). The glial scar and central nervous system repair. Brain Res Bull.

[12] Faulkner JR, Herrmann JE, Woo MJ, Tansey KE, Doan NB, Sofroniew MV (2004). Reactive astrocytes protect tissue and preserve function after spinal cord injury. J Neurosci.

[13] Krause JS, Sternberg M, Lottes S, Maides J (1997). Mortality after spinal cord injury: an 11-year prospective study. Arch Phys Med Rehabil.

[14] Ahuja CS, Wilson JR, Nori S, Kotter MRN, Druschel C, Curt A (2017). Traumatic spinal cord injury. Nat Rev Dis Primers.

[15] Alexander JK, Popovich PG (2009). Neuroinflammation in spinal cord injury: therapeutic targets for neuroprotection and regeneration. Prog Brain Res.

[16] Donnelly DJ, Popovich PG (2008). Inflammation and its role in neuroprotection, axonal regeneration and functional recovery after spinal cord injury. Exp Neurol.

[17] Jones TB, McDaniel EE, Popovich PG (2005). Inflammatory-mediated injury and repair in the traumatically injured spinal cord. Curr Pharm Des.

[18] Trivedi A, Olivas AD, Noble-Haeusslein LJ (2006). Inflammation and Spinal Cord Injury: Infiltrating Leukocytes as Determinants of Injury and Repair Processes. Clin Neurosci Res.

[19] Knerlich-Lukoschus F, Held-Feindt J (2015). Chemokine-ligands/receptors: multiplayers in traumatic spinal cord injury. Mediators Inflamm.

[20] Belperio JA, Keane MP, Arenberg DA, Addison CL, Ehlert JE, Burdick MD (2000). CXC chemokines in angiogenesis. J Leukoc Biol.

[21] Lee EY, Lee ZH, Song YW (2009). CXCL10 and autoimmune diseases. Autoimmun Rev.

[22] Gonzalez R, Hickey MJ, Espinosa JM, Nistor G, Lane TE, Keirstead HS (2007). Therapeutic neutralization of CXCL10 decreases secondary degeneration and functional deficit after spinal cord injury in mice. Regen Med.

[23] Mordillo-Mateos L, Sanchez-Ramos A, Coperchini F, Bustos-Guadamillas I, Alonso-Bonilla C, Vargas-Baquero E (2019). Development of chronic pain in males with traumatic spinal cord injury: role of circulating levels of the chemokines CCL2 and CXCL10 in subacute stage. Spinal Cord.

[24] Glaser J, Gonzalez R, Perreau VM, Cotman CW, Keirstead HS (2004). Neutralization of the chemokine CXCL10 enhances tissue sparing and angiogenesis following spinal cord injury. J Neurosci Res.

[25] Glaser J, Gonzalez R, Sadr E, Keirstead HS (2006). Neutralization of the chemokine CXCL10 reduces apoptosis and increases axon sprouting after spinal cord injury. Journal of neuroscience research.

[26] Kuang Y, Wu Y, Jiang H, Wu D (1996). Selective G protein coupling by C-C chemokine receptors. J Biol Chem.

[27] Fernandez EJ, Lolis E (2002). Structure, function, and inhibition of chemokines. Annu Rev Pharmacol Toxicol.

[28] Williams JL, Holman DW, Klein RS (2014). Chemokines in the balance: maintenance of homeostasis and protection at CNS barriers. Front Cell Neurosci.

[29] Antonelli A, Ferrari SM, Giuggioli D, Ferrannini E, Ferri C, Fallahi P (2014). Chemokine (C-X-C motif) ligand (CXCL)10 in autoimmune diseases. Autoimmun Rev.

[30] Strieter RM, Polverini PJ, Kunkel SL, Arenberg DA, Burdick MD, Kasper J (1995). The functional role of the ELR motif in CXC chemokine-mediated angiogenesis. J Biol Chem.

[31] Gao J, Wu L, Wang S, Chen X (2020). Role of Chemokine (C-X-C Motif) Ligand 10 (CXCL10) in Renal Diseases. Mediators Inflamm.

[32] Gasperini S, Marchi M, Calzetti F, Laudanna C, Vicentini L, Olsen H (1999). Gene expression and production of the monokine induced by IFN-gamma (MIG), IFN-inducible T cell alpha chemoattractant (I-TAC), and IFN-gamma-inducible protein-10 (IP-10) chemokines by human neutrophils. J Immunol.

[33] Dajotoy T, Andersson P, Bjartell A, Lofdahl CG, Tapper H, Egesten A (2004). Human eosinophils produce the T cell-attracting chemokines MIG and IP-10 upon stimulation with IFN-gamma. J Leukoc Biol.

[34] Sauty A, Dziejman M, Taha RA, Iarossi AS, Neote K, Garcia-Zepeda EA (1999). The T cell-specific CXC chemokines IP-10, Mig, and I-TAC are expressed by activated human bronchial epithelial cells. J Immunol.

[35] Ohmori Y, Hamilton TA (1995). The interferon-stimulated response element and a kappa B site mediate synergistic induction of murine IP-10 gene transcription by IFN-gamma and TNF-alpha. J Immunol.

[36] Qian C, An H, Yu Y, Liu S, Cao X (2007). TLR agonists induce regulatory dendritic cells to recruit Th1 cells via preferential IP-10 secretion and inhibit Th1 proliferation. Blood.

[37] Ohmori Y, Wyner L, Narumi S, Armstrong D, Stoler M, Hamilton TA (1993). Tumor necrosis factor-alpha induces cell type and tissue-specific expression of chemoattractant cytokines in vivo. Am J Pathol.

[38] Luster AD, Jhanwar SC, Chaganti RS, Kersey JH, Ravetch JV (1987). Interferon-inducible gene maps to a chromosomal band associated with a (4;11) translocation in acute leukemia cells. Proc Natl Acad Sci U S A.

[39] Loetscher M, Gerber B, Loetscher P, Jones SA, Piali L, Clark-Lewis I (1996). Chemokine receptor specific for IP10 and mig: structure, function, and expression in activated T-lymphocytes. J Exp Med.

[40] Berchiche YA, Sakmar TP (2016). CXC Chemokine Receptor 3 Alternative Splice Variants Selectively Activate Different Signaling Pathways. Mol Pharmacol.

[41] Kuo PT, Zeng Z, Salim N, Mattarollo S, Wells JW, Leggatt GR (2018). The Role of CXCR3 and Its Chemokine Ligands in Skin Disease and Cancer. Front Med (Lausanne).

[42] Rappert A, Bechmann I, Pivneva T, Mahlo J, Biber K, Nolte C (2004). CXCR3-dependent microglial recruitment is essential for dendrite loss after brain lesion. The Journal of neuroscience: the official journal of the Society for Neuroscience.

[43] Xia MQ, Bacskai BJ, Knowles RB, Qin SX, Hyman BT (2000). Expression of the chemokine receptor CXCR3 on neurons and the elevated expression of its ligand IP-10 in reactive astrocytes: in vitro ERK1/2 activation and role in Alzheimer's disease. J Neuroimmunol.

[44] Zhu Y, Vergote D, Pardo C, Noorbakhsh F, McArthur JC, Hollenberg MD (2009). CXCR3 activation by lentivirus infection suppresses neuronal autophagy: neuroprotective effects of antiretroviral therapy. FASEB J.

[45] Wu Q, Dhir R, Wells A (2012). Altered CXCR3 isoform expression regulates prostate cancer cell migration and invasion. Mol Cancer.

[46] Ji R, Lee CM, Gonzales LW, Yang Y, Aksoy MO, Wang P (2008). Human type II pneumocyte chemotactic responses to CXCR3 activation are mediated by splice variant A. Am J Physiol Lung Cell Mol Physiol.

[47] Lasagni L, Francalanci M, Annunziato F, Lazzeri E, Giannini S, Cosmi L (2003). An alternatively spliced variant of CXCR3 mediates the inhibition of endothelial cell growth induced by IP-10, Mig, and I-TAC, and acts as functional receptor for platelet factor 4. J Exp Med.

[48] Bodnar RJ, Yates CC, Wells A (2006). IP-10 blocks vascular endothelial growth factor-induced endothelial cell motility and tube formation via inhibition of calpain. Circ Res.

[49] Bodnar RJ, Yates CC, Rodgers ME, Du X, Wells A (2009). IP-10 induces dissociation of newly formed blood vessels. J Cell Sci.

[50] Yates-Binder CC, Rodgers M, Jaynes J, Wells A, Bodnar RJ, Turner T (2012). An IP-10 (CXCL10)-derived peptide inhibits angiogenesis. PLoS One.

[51] Leloup L, Shao H, Bae YH, Deasy B, Stolz D, Roy P (2010). m-Calpain activation is regulated by its membrane localization and by its binding to phosphatidylinositol 4,5-bisphosphate. J Biol Chem.

[52] Petrai I, Rombouts K, Lasagni L, Annunziato F, Cosmi L, Romanelli RG (2008). Activation of p38(MAPK) mediates the angiostatic effect of the chemokine receptor CXCR3-B. Int J Biochem Cell Biol.

[53] Balan M, Pal S (2014). A novel CXCR3-B chemokine receptor-induced growth-inhibitory signal in cancer cells is mediated through the regulation of Bach-1 protein and Nrf2 protein nuclear translocation. J Biol Chem.

[54] Tokunaga R, Zhang W, Naseem M, Puccini A, Berger MD, Soni S (2018). CXCL9, CXCL10, CXCL11/CXCR3 axis for immune activation - A target for novel cancer therapy. Cancer Treat Rev.

[55] Chen Y, Yin D, Fan B, Zhu X, Chen Q, Li Y (2019). Chemokine CXCL10/CXCR3 signaling contributes to neuropathic pain in spinal cord and dorsal root ganglia after chronic constriction injury in rats. Neurosci Lett.

[56] Yu Q, Tian DL, Tian Y, Zhao XT, Yang XY (2018). Elevation of the Chemokine Pair CXCL10/CXCR3 Initiates Sequential Glial Activation and Crosstalk During the Development of Bimodal Inflammatory Pain after Spinal Cord Ischemia Reperfusion. Cell Physiol Biochem.

[57] Antonelli A, Ferri C, Ferrari SM, Colaci M, Fallahi P (2008). Immunopathogenesis of HCV-related endocrine manifestations in chronic hepatitis and mixed cryoglobulinemia. Autoimmun Rev.

[58] Nakajima C, Mukai T, Yamaguchi N, Morimoto Y, Park WR, Iwasaki M (2002). Induction of the chemokine receptor CXCR3 on TCR-stimulated T cells: dependence on the release from persistent TCR-triggering and requirement for IFN-gamma stimulation. Eur J Immunol.

[59] Taub DD, Lloyd AR, Conlon K, Wang JM, Ortaldo JR, Harada A (1993). Recombinant human interferon-inducible protein 10 is a chemoattractant for human monocytes and T lymphocytes and promotes T cell adhesion to endothelial cells. J Exp Med.

[60] Klein RS, Lin E, Zhang B, Luster AD, Tollett J, Samuel MA (2005). Neuronal CXCL10 directs CD8+ T-cell recruitment and control of West Nile virus encephalitis. J Virol.

[61] Lindell DM, Lane TE, Lukacs NW (2008). CXCL10/CXCR3-mediated responses promote immunity to respiratory syncytial virus infection by augmenting dendritic cell and CD8(+) T cell efficacy. Eur J Immunol.

[62] Sidahmed AM, Leon AJ, Bosinger SE, Banner D, Danesh A, Cameron MJ (2012). CXCL10 contributes to p38-mediated apoptosis in primary T lymphocytes in vitro. Cytokine.

[63] Kvestak D, Juranic Lisnic V, Lisnic B, Tomac J, Golemac M, Brizic I (2021). NK/ILC1 cells mediate neuroinflammation and brain pathology following congenital CMV infection. J Exp Med.

[64] Jinquan T, Jing C, Jacobi HH, Reimert CM, Millner A, Quan S (2000). CXCR3 expression and activation of eosinophils: role of IFN-gamma-inducible protein-10 and monokine induced by IFN-gamma. J Immunol.

[65] Gao N, Liu X, Wu J, Li J, Dong C, Wu X (2017). CXCL10 suppression of hem- and lymph-angiogenesis in inflamed corneas through MMP13. Angiogenesis.

[66] Angiolillo AL, Sgadari C, Taub DD, Liao F, Farber JM, Maheshwari S (1995). Human interferon-inducible protein 10 is a potent inhibitor of angiogenesis in vivo. The Journal of experimental medicine.

[67] Sanders VJ, Pittman CA, White MG, Wang G, Wiley CA, Achim CL (1998). Chemokines and receptors in HIV encephalitis. AIDS.

[68] Cheeran MCJ, Hu S, Sheng WS, Peterson PK, Lokensgard JR (2003). CXCL10 production from cytomegalovirus-stimulated microglia is regulated by both human and viral interleukin-10. J Virol.

[69] Lafon M, Megret F, Lafage M, Prehaud C (2006). The innate immune facet of brain: human neurons express TLR-3 and sense viral dsRNA. J Mol Neurosci.

[70] van Marle G, Henry S, Todoruk T, Sullivan A, Silva C, Rourke SB (2004). Human immunodeficiency virus type 1 Nef protein mediates neural cell death: a neurotoxic role for IP-10. Virology.

[71] Wilson NO, Jain V, Roberts CE, Lucchi N, Joel PK, Singh MP (2011). CXCL4 and CXCL10 predict risk of fatal cerebral malaria. Dis Markers.

[72] Harbuzariu A, Pitts S, Cespedes JC, Harp KO, Nti A, Shaw AP (2019). Modelling heme-mediated brain injury associated with cerebral malaria in human brain cortical organoids. Sci Rep.

[73] Mehla R, Bivalkar-Mehla S, Nagarkatti M, Chauhan A (2012). Programming of neurotoxic cofactor CXCL-10 in HIV-1-associated dementia: abrogation of CXCL-10-induced neuro-glial toxicity in vitro by PKC activator. J Neuroinflamm.

[74] Sui Y, Stehno-Bittel L, Li S, Loganathan R, Dhillon NK, Pinson D (2006). CXCL10-induced cell death in neurons: role of calcium dysregulation. Eur J Neurosci.

[75] de Haas AH, van Weering HR, de Jong EK, Boddeke HW, Biber KP (2007). Neuronal chemokines: versatile messengers in central nervous system cell interaction. Mol Neurobiol.

[76] Koper OM, Kamińska J, Sawicki K, Kemona H (2018). CXCL9, CXCL10, CXCL11, and their receptor (CXCR3) in neuroinflammation and neurodegeneration. Adv Clin Exp Med.

[77] Gomez Perdiguero E, Klapproth K, Schulz C, Busch K, Azzoni E, Crozet L (2015). Tissue-resident macrophages originate from yolk-sac-derived erythro-myeloid progenitors. Nature.

[78] Greenmyer JR, Gaultney RA, Brissette CA, Watt JA (2018). Primary Human Microglia Are Phagocytically Active and Respond to Borrelia burgdorferi With Upregulation of Chemokines and Cytokines. Front Microbiol.

[79] Hua LL, Lee SC (2000). Distinct patterns of stimulus-inducible chemokine mRNA accumulation in human fetal astrocytes and microglia. Glia.

[80] Mayer AMS, Murphy J, MacAdam D, Osterbauer C, Baseer I, Hall ML (2016). Classical and Alternative Activation of Cyanobacterium Oscillatoria sp. Lipopolysaccharide-Treated Rat Microglia in vitro. Toxicol Sci.

[81] Woo J, Han D, Wang JI, Park J, Kim H, Kim Y (2017). Quantitative Proteomics Reveals Temporal Proteomic Changes in Signaling Pathways during BV2 Mouse Microglial Cell Activation. J Proteome Res.

[82] Müller M, Carter S, Hofer MJ, Campbell IL (2010). Review: The chemokine receptor CXCR3 and its ligands CXCL9, CXCL10 and CXCL11 in neuroimmunity-a tale of conflict and conundrum. Neuropathol Appl Neurobiol.

[83] Kawai T, Takeuchi O, Fujita T, Inoue J, Muhlradt PF, Sato S (2001). Lipopolysaccharide stimulates the MyD88-independent pathway and results in activation of IFN-regulatory factor 3 and the expression of a subset of lipopolysaccharide-inducible genes. J Immunol.

[84] Kielian T (2006). Toll-like receptors in central nervous system glial inflammation and homeostasis. J Neurosci Res.

[85] Shen Q, Zhang R, Bhat NR (2006). MAP kinase regulation of IP10/CXCL10 chemokine gene expression in microglial cells. Brain Res.

[86] Tong W, Hu ZY, Sun GY (1995). Stimulation of group II phospholipase A2 mRNA expression and release in an immortalized astrocyte cell line (DITNC) by LPS, TNF alpha, and IL-1 beta. Interactive effects. Mol Chem Neuropathol.

[87] Brissette CA, Kees ED, Burke MM, Gaultney RA, Floden AM, Watt JA (2013). The multifaceted responses of primary human astrocytes and brain microvascular endothelial cells to the Lyme disease spirochete, Borrelia burgdorferi. ASN Neuro.

[88] Jack CS, Arbour N, Manusow J, Montgrain V, Blain M, McCrea E (2005). TLR signaling tailors innate immune responses in human microglia and astrocytes. J Immunol.

[89] Bhowmick S, Duseja R, Das S, Appaiahgiri MB, Vrati S, Basu A (2007). Induction of IP-10 (CXCL10) in astrocytes following Japanese encephalitis. Neurosci Lett.

[90] Stefanik M, Formanova P, Bily T, Vancova M, Eyer L, Palus M (2018). Characterisation of Zika virus infection in primary human astrocytes. BMC Neurosci.

[91] Liu C, Cui G, Zhu M, Kang X, Guo H (2014). Neuroinflammation in Alzheimer's disease: chemokines produced by astrocytes and chemokine receptors. Int J Clin Exp Pathol.

[92] Omari KM, John GR, Sealfon SC, Raine CS (2005). CXC chemokine receptors on human oligodendrocytes: implications for multiple sclerosis. Brain.

[93] Ransohoff RM, Hamilton TA, Tani M, Stoler MH, Shick HE, Major JA (1993). Astrocyte expression of mRNA encoding cytokines IP-10 and JE/MCP-1 in experimental autoimmune encephalomyelitis. FASEB J.

[94] Tani M, Glabinski AR, Tuohy VK, Stoler MH, Estes ML, Ransohoff RM (1996). In situ hybridization analysis of glial fibrillary acidic protein mRNA reveals evidence of biphasic astrocyte activation during acute experimental autoimmune encephalomyelitis. Am J Pathol.

[95] Godiska R, Chantry D, Dietsch GN, Gray PW (1995). Chemokine expression in murine experimental allergic encephalomyelitis. J Neuroimmunol.

[96] Fife BT, Kennedy KJ, Paniagua MC, Lukacs NW, Kunkel SL, Luster AD (2001). CXCL10 (IFN-gamma-inducible protein-10) control of encephalitogenic CD4+ T cell accumulation in the central nervous system during experimental autoimmune encephalomyelitis. J Immunol.

[97] Biber K, Dijkstra I, Trebst C, De Groot CJA, Ransohoff RM, Boddeke HWGM (2002). Functional expression of CXCR3 in cultured mouse and human astrocytes and microglia. Neuroscience.

[98] Flynn G, Maru S, Loughlin J, Romero IA, Male D (2003). Regulation of chemokine receptor expression in human microglia and astrocytes. J Neuroimmunol.

[99] Biber K, Sauter A, Brouwer N, Copray SC, Boddeke HW (2001). Ischemia-induced neuronal expression of the microglia attracting chemokine Secondary Lymphoid-tissue Chemokine (SLC). Glia.

[100] Rappert A, Biber K, Nolte C, Lipp M, Schubel A, Lu B (2002). Secondary lymphoid tissue chemokine (CCL21) activates CXCR3 to trigger a Cl- current and chemotaxis in murine microglia. Journal of immunology (Baltimore, Md: 1950).

[101] Krauthausen M, Saxe S, Zimmermann J, Emrich M, Heneka MT, Muller M (2014). CXCR3 modulates glial accumulation and activation in cuprizone-induced demyelination of the central nervous system. J Neuroinflammation.

[102] Wilson NO, Solomon W, Anderson L, Patrickson J, Pitts S, Bond V (2013). Pharmacologic inhibition of CXCL10 in combination with anti-malarial therapy eliminates mortality associated with murine model of cerebral malaria. PLoS One.

[103] Balabanov R, Strand K, Goswami R, McMahon E, Begolka W, Miller SD (2007). Interferon-gamma-oligodendrocyte interactions in the regulation of experimental autoimmune encephalomyelitis. The Journal of neuroscience: the official journal of the Society for Neuroscience.

[104] Carter SL, Müller M, Manders PM, Campbell IL (2007). Induction of the genes for Cxcl9 and Cxcl10 is dependent on IFN-gamma but shows differential cellular expression in experimental autoimmune encephalomyelitis and by astrocytes and microglia in vitro. Glia.

[105] Nash B, Thomson CE, Linington C, Arthur AT, McClure JD, McBride MW (2011). Functional duality of astrocytes in myelination. J Neurosci.

[106] Sorensen TL, Trebst C, Kivisakk P, Klaege KL, Majmudar A, Ravid R (2002). Multiple sclerosis: a study of CXCL10 and CXCR3 co-localization in the inflamed central nervous system. J Neuroimmunol.

[107] Ahuja CS, Fehlings M (2016). Concise Review: Bridging the Gap: Novel Neuroregenerative and Neuroprotective Strategies in Spinal Cord Injury. Stem Cells Transl Med.

[108] Guha A, Tator CH, Rochon J (1989). Spinal cord blood flow and systemic blood pressure after experimental spinal cord injury in rats. Stroke.

[109] Guha A, Tator CH (1988). Acute cardiovascular effects of experimental spinal cord injury. J Trauma.

[110] Whetstone WD, Hsu J-YC, Eisenberg M, Werb Z, Noble-Haeusslein LJ (2003). Blood-spinal cord barrier after spinal cord injury: relation to revascularization and wound healing. Journal of neuroscience research.

[111] LaPlaca MC, Simon CM, Prado GR, Cullen DK (2007). CNS injury biomechanics and experimental models. Progress in brain research.

[112] Choo AM, Liu J, Lam CK, Dvorak M, Tetzlaff W, Oxland TR (2007). Contusion, dislocation, and distraction: primary hemorrhage and membrane permeability in distinct mechanisms of spinal cord injury. J Neurosurg Spine.

[113] Popovich PG, Wei P, Stokes BT (1997). Cellular inflammatory response after spinal cord injury in Sprague-Dawley and Lewis rats. J Comp Neurol.

[114] Kwon BK, Stammers AMT, Belanger LM, Bernardo A, Chan D, Bishop CM (2010). Cerebrospinal fluid inflammatory cytokines and biomarkers of injury severity in acute human spinal cord injury. J Neurotrauma.

[115] Davies AL, Hayes KC, Dekaban GA (2007). Clinical correlates of elevated serum concentrations of cytokines and autoantibodies in patients with spinal cord injury. Archives of physical medicine and rehabilitation.

[116] Bank M, Stein A, Sison C, Glazer A, Jassal N, McCarthy D (2015). Elevated circulating levels of the pro-inflammatory cytokine macrophage migration inhibitory factor in individuals with acute spinal cord injury. Archives of physical medicine and rehabilitation.

[117] Liu S-q, Ma Y-g, Peng H, Fan L (2005). Monocyte chemoattractant protein-1 level in serum of patients with acute spinal cord injury. Chin J Traumatol.

[118] Casha S, Rice T, Stirling DP, Silva C, Gnanapavan S, Giovannoni G (2018). Cerebrospinal Fluid Biomarkers in Human Spinal Cord Injury from a Phase II Minocycline Trial. J Neurotrauma.

[119] Li S, Mealing GA, Morley P, Stys PK (1999). Novel injury mechanism in anoxia and trauma of spinal cord white matter: glutamate release via reverse Na+-dependent glutamate transport. The Journal of neuroscience: the official journal of the Society for Neuroscience.

[120] Huang W, Vodovotz Y, Kusturiss MB, Barclay D, Greenwald K, Boninger ML (2014). Identification of distinct monocyte phenotypes and correlation with circulating cytokine profiles in acute response to spinal cord injury: a pilot study. PM R.

[121] Norenberg MD, Smith J, Marcillo A (2004). The pathology of human spinal cord injury: defining the problems. J Neurotrauma.

[122] Tator CH (1995). Update on the pathophysiology and pathology of acute spinal cord injury. Brain Pathol.

[123] McKeon RJ, Schreiber RC, Rudge JS, Silver J (1991). Reduction of neurite outgrowth in a model of glial scarring following CNS injury is correlated with the expression of inhibitory molecules on reactive astrocytes. The Journal of neuroscience: the official journal of the Society for Neuroscience.

[124] McTigue DM, Tani M, Krivacic K, Chernosky A, Kelner GS, Maciejewski D (1998). Selective chemokine mRNA accumulation in the rat spinal cord after contusion injury. J Neurosci Res.

[125] Rice T, Larsen J, Rivest S, Yong VW (2007). Characterization of the early neuroinflammation after spinal cord injury in mice. Journal of neuropathology and experimental neurology.

[126] Wu X-B, He L-N, Jiang B-C, Shi H, Bai X-Q, Zhang W-W (2018). Spinal CXCL9 and CXCL11 are not involved in neuropathic pain despite an upregulation in the spinal cord following spinal nerve injury. Mol Pain.

[127] Israelsson C, Bengtsson H, Lobell A, Nilsson LNG, Kylberg A, Isaksson M (2010). Appearance of Cxcl10-expressing cell clusters is common for traumatic brain injury and neurodegenerative disorders. Eur J Neurosci.

[128] Garcia E, Aguilar-Cevallos J, Silva-Garcia R, Ibarra A (2016). Cytokine and Growth Factor Activation In Vivo and In Vitro after Spinal Cord Injury. Mediators Inflamm.

[129] Hassanshahi G, Amin M, Shunmugavel A, Vazirinejad R, Vakilian A, Sanji M (2013). Temporal expression profile of CXC chemokines in serum of patients with spinal cord injury. Neurochem Int.

[130] Leister I, Haider T, Mattiassich G, Kramer JLK, Linde LD, Pajalic A (2020). Biomarkers in Traumatic Spinal Cord Injury-Technical and Clinical Considerations: A Systematic Review. Neurorehabil Neural Repair.

[131] Morris G, Fernandes BS, Puri BK, Walker AJ, Carvalho AF, Berk M (2018). Leaky brain in neurological and psychiatric disorders: Drivers and consequences. Aust N Z J Psychiatry.

[132] David S, Zarruk JG, Ghasemlou N (2012). Inflammatory pathways in spinal cord injury. Int Rev Neurobiol.

[133] Popovich PG, Stokes BT, Whitacre CC (1996). Concept of autoimmunity following spinal cord injury: possible roles for T lymphocytes in the traumatized central nervous system. J Neurosci Res.

[134] Liu MT, Keirstead HS, Lane TE (2001). Neutralization of the chemokine CXCL10 reduces inflammatory cell invasion and demyelination and improves neurological function in a viral model of multiple sclerosis. Journal of immunology (Baltimore, Md: 1950).

[135] Dumont RJ, Okonkwo DO, Verma S, Hurlbert RJ, Boulos PT, Ellegala DB (2001). Acute spinal cord injury, part I: pathophysiologic mechanisms. Clin Neuropharmacol.

[136] Fee D, Crumbaugh A, Jacques T, Herdrich B, Sewell D, Auerbach D (2003). Activated/effector CD4+ T cells exacerbate acute damage in the central nervous system following traumatic injury. Journal of neuroimmunology.

[137] Hu J, Ma X, Lindner DJ, Karra S, Hofmann ER, Reddy SP (2001). Modulation of p53 dependent gene expression and cell death through thioredoxin-thioredoxin reductase by the Interferon-Retinoid combination. Oncogene.

[138] Finnerup NB, Johannesen IL, Sindrup SH, Bach FW, Jensen TS (2001). Pain and dysesthesia in patients with spinal cord injury: A postal survey. Spinal cord.

[139] Werhagen L, Budh CN, Hultling C, Molander C (2004). Neuropathic pain after traumatic spinal cord injury-relations to gender, spinal level, completeness, and age at the time of injury. Spinal cord.

[140] Singh R, Dhankar SS, Rohilla R (2008). Quality of life of people with spinal cord injury in Northern India. Int J Rehabil Res.

[141] Finnerup NB, Jensen TS (2004). Spinal cord injury pain-mechanisms and treatment. Eur J Neurol.

[142] Cardenas DD, Felix ER (2009). Pain after spinal cord injury: a review of classification, treatment approaches, and treatment assessment. PM & R: the journal of injury, function, and rehabilitation.

[143] Zhang Q, Xiao Z, Huang C, Hu S, Kulkarni P, Martinez E (2018). Local field potential decoding of the onset and intensity of acute pain in rats. Sci Rep.

[144] Vogt BA (2005). Pain and emotion interactions in subregions of the cingulate gyrus. Nat Rev Neurosci.

[145] Johansen JP, Fields HL (2004). Glutamatergic activation of anterior cingulate cortex produces an aversive teaching signal. Nat Neurosci.

[146] Jiang B-C, He L-N, Wu X-B, Shi H, Zhang W-W, Zhang Z-J (2017). Promoted Interaction of C/EBPα with Demethylated Cxcr3 Gene Promoter Contributes to Neuropathic Pain in Mice. The Journal of neuroscience: the official journal of the Society for Neuroscience.

[147] Qin J, Li A, Huang Y, Teng R-H, Yang Y, Yao Y-X (2020). CXCR3 contributes to neuropathic pain via ERK activation in the anterior cingulate cortex. Biochem Biophys Res Commun.

[148] Piotrowska A, Rojewska E, Pawlik K, Kreiner G, Ciechanowska A, Makuch W (2018). Pharmacological blockade of CXCR3 by (±)-NBI-74330 reduces neuropathic pain and enhances opioid effectiveness - Evidence from in vivo and in vitro studies. Biochim Biophys Acta Mol Basis Dis.

[149] Rush PJ (1989). The rheumatic manifestations of traumatic spinal cord injury. Semin Arthritis Rheum.

[150] van Kuijk AA, Geurts ACH, van Kuppevelt HJM (2002). Neurogenic heterotopic ossification in spinal cord injury. Spinal cord.

[151] Lee J-H, Kim B, Jin WJ, Kim H-H, Ha H, Lee ZH (2017). Pathogenic roles of CXCL10 signaling through CXCR3 and TLR4 in macrophages and T cells: relevance for arthritis. Arthritis Res Ther.

[152] Samson G, Cardenas DD (2007). Neurogenic bladder in spinal cord injury. Phys Med Rehabil Clin N Am.

[153] Tyagi P, Kadekawa K, Kashyap M, Pore S, Yoshimura N (2016). Spontaneous Recovery of Reflex Voiding Following Spinal Cord Injury Mediated by Anti-inflammatory and Neuroprotective Factors. Urology.

[154] Sezer N, Akkus S, Ugurlu FG (2015). Chronic complications of spinal cord injury. World J Orthop.

[155] Krishnan S, Karg PE, Boninger ML, Vodovotz Y, Constantine G, Sowa GA (2016). Early Detection of Pressure Ulcer Development Following Traumatic Spinal Cord Injury Using Inflammatory Mediators. Archives of physical medicine and rehabilitation.

[156] Ahuja CS, Nori S, Tetreault L, Wilson J, Kwon B, Harrop J (2017). Traumatic Spinal Cord Injury-Repair and Regeneration. Neurosurgery.

[157] Putatunda R, Bethea JR, Hu WH (2018). Potential immunotherapies for traumatic brain and spinal cord injury. Chin J Traumatol.

[158] Ghirnikar RS, Lee YL, Eng LF (2001). Chemokine antagonist infusion promotes axonal sparing after spinal cord contusion injury in rat. Journal of neuroscience research.

[159] Fan B, Wei Z, Yao X, Shi G, Cheng X, Zhou X (2018). Microenvironment Imbalance of Spinal Cord Injury. Cell Transplant.

[160] Begenisic T, Pavese C, Aiachini B, Nardone A, Rossi D (2021). Dynamics of biomarkers across the stages of traumatic spinal cord injury - implications for neural plasticity and repair. Restor Neurol Neurosci.

[161] Leister I, Haider T, Mattiassich G, Kramer JLK, Linde LD, Pajalic A (2020). Biomarkers in Traumatic Spinal Cord Injury-Technical and Clinical Considerations: A Systematic Review. Neurorehabil Neural Repair.

[162] Shechter R, London A, Varol C, Raposo C, Cusimano M, Yovel G (2009). Infiltrating blood-derived macrophages are vital cells playing an anti-inflammatory role in recovery from spinal cord injury in mice. PLoS Med.

[163] Hines DJ, Hines RM, Mulligan SJ, Macvicar BA (2009). Microglia processes block the spread of damage in the brain and require functional chloride channels. Glia.

[164] Anderson MA, Burda JE, Ren Y, Ao Y, O'Shea TM, Kawaguchi R (2016). Astrocyte scar formation aids central nervous system axon regeneration. Nature.

